# Synthesis and Promising *in Vitro* Antiproliferative Activity of Sulfones of a 5-Nitrothiazole Series

**DOI:** 10.3390/molecules18010097

**Published:** 2012-12-21

**Authors:** Anita Cohen, Maxime D. Crozet, Pascal Rathelot, Nadine Azas, Patrice Vanelle

**Affiliations:** 1 Laboratoire de Pharmaco-Chimie Radicalaire, Faculté de Pharmacie, Institut de Chimie Radicalaire ICR UMR 7273, Aix-Marseille Univ, CNRS, 27 Boulevard Jean Moulin - CS30064 - 13385 Marseille cedex 05, France; 2 Infections Parasitaires, Transmission, Pharmacologie et Thérapeutique IP-TPT UMR MD3, Faculté de Pharmacie, Aix-Marseille Univ, 27 Boulevard Jean Moulin - CS30064 - 13385 Marseille cedex 05, France

**Keywords:** 5-nitrothiazole, sulfones, microwave irradiation, *in vitro* antiproliferative, HepG2 cell line, activity cellular specificity

## Abstract

The synthesis in water of new sulfone derivatives under microwave irradiation is described. This eco-friendly process leads to the expected products in good yields by reaction of various substituted sulfinates (commercially available or obtained by reduction of the corresponding sulfonyl chlorides) with 4-chloromethyl-2-methyl-5-nitro-1,3-thiazole. In order to evaluate the antiproliferative effect of these compounds, several sulfone derivatives are also dichlorinated on the Cα next to the sulfonyl group. An evaluation on different cancer cell lines reveals promising selective *in vitro* antiproliferative activity toward HepG2 human cell lines by dihydrogenated sulfones, suggesting further research should be to explore their anticancer potential in the treatment of liver cancer.

## 1. Introduction

Recent years have seen major advances in research and development concerning new small molecules whose antiproliferative activity appears promising for the treatment of cancer [1,2]. Among chemical compounds already developed as potential anticancer agents, some sulfones [3,4] such as celecoxib [5], and heterocyclic molecules such as thiazole derivatives [6,7], have recently been reported to display promising antiproliferative activity. 

Thiazoles are one of the most prevalent heterocyclic nuclei, among compounds displaying biological activities [8], such as β-lactams [9,10,11], urate-lowering drugs (febuxostat) [12], or antiparasitic agents [13,14,15]. Furthermore, sulfonylmethyl groups are well-known to be useful in synthetic methodologies and they can be used further for the preparation of various functionalized products. For example, the expected acidity of the C-Hα next to sulfonyl groups offers the opportunity to carry out various reactions at this position [16,17,18].

In continuation of our research program centered on the design and synthesis of novel molecules, we focused our work on the synthesis and the evaluation of some new heterocyclic compounds displaying diverse biological activities [19,20,21,22,23,24]. In this context, we decided to explore the antiproliferative potential of new sulfonyl derivatives in the 5-nitro-1,3-thiazole series. We report herein the synthesis of such molecules from the reaction in water of 4-chloromethyl-2-methyl-5-nitro-1,3-thiazole (**1**) with various sulfinate anions under microwave irradiation. This is in continuation of our research program directed towards the study of electron transfer reactions in heterocyclic series [25,26] and microwave-assisted [27,28] eco-friendly processes [29,30]. The antiproliferative activity both of sulfones and of some α-dichlorinated sulfonyl derivatives was comparatively evaluated on the CHO and HepG2 cell lines, and results confirmed the promising antiproliferative effect of dihydrogenated sulfones towards the HepG2 cell line.

## 2. Results and Discussion

### 2.1. Chemistry

**Scheme 1:**
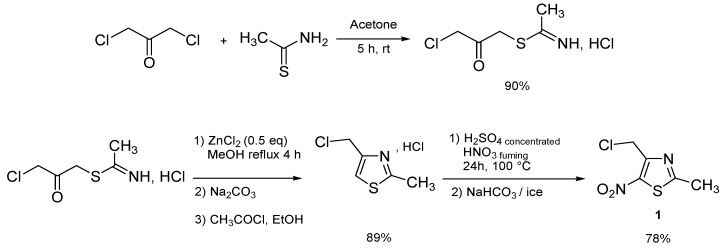
Preparation of 4-chloromethyl-2-methyl-5-nitro-1,3-thiazole (**1**) [32].

The required starting material, 4-chloromethyl-2-methyl-5-nitro-1,3-thiazole (**1**), was prepared in 62% overall yield by sequential condensation between 1,3-dichloroacetone with thioacetamide [31], cyclization using ZnCl_2_ in refluxing methanol, and nitration of 2-methyl-4-chloromethyl-1,3-thiazole hydrochloride [32] (Scheme 1).

The synthesis of 2-methyl-5-nitro-4-phenylsulfonylmethyl-1,3-thiazole (**2a**) by reaction of **1** with sodium phenylsulfinate in anhydrous methanol, under S_RN_1 conditions [33,34] (inert atmosphere (Ar) and 60 W lamp irradiation), at room temperature (rt) for 24 h [32] has already been described. Based on a number of reports suggesting that chemical reactions using water as a solvent in conjunction with microwave heating [35,36,37] were more eco-friendly, we adapted this alternative method to the synthesis of **2a**. Water is an attractive alternative to traditional organic solvents due to its practical advantages: it is inexpensive, non-flammable, non-toxic, and environmentally sustainable as it removes the problem of pollution by organic solvents. Water has also proven to be an excellent solvent for microwave-promoted synthesis [38,39,40]. Furthermore, as well as being energy efficient, microwaves can also enhance reaction rates, and in many cases, improve yields [41,42,43,44].

**Scheme 2:**
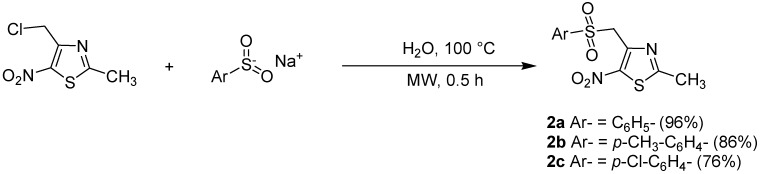
Preparation of sulfones **2a**, **2b**, **2c** by reaction of the corresponding commercialized sulfinate salts with 4-chloromethyl-2-methyl-5-nitro-1,3-thiazole (**1**).

Based on previous results for reduction reaction using microwave promotion, an initial irradiation of 500 W at a temperature of 100 °C was applied [45]. We subsequently tried reducing the irradiation power, finally establishing that the optimal experimental conditions to carry out the reactions under microwave irradiation were 200 W and a temperature held at 100 °C until the completion of the reaction (Scheme 2). In order to evaluate the efficiency of these latter experimental conditions *versus* classical heating as previously described, we compared the synthesis of 2-methyl-5-nitro-4-phenylsulfonylmethyl-1,3-thiazole (**2a**) by the two methods (classical heating in methanol [32] *versus* microwave irradiation in water). As expected, it proved more convenient to carry out the reaction in water and using microwave technology (Table 1 entry 1). Indeed, it took only 30 min to complete the reaction with an excellent yield (96%) under these conditions, while the classical heating conditions required a reaction time of 24 h to synthesize **2a** in a lower yield (84%). The reaction rate was thus, accelerated up to 48 times, and led to higher yields.

Following these excellent first results, we extended the study to *p*-tosyl and *p*-chlorophenyl sulfinate anions, with a view to exploring the chemical and biological influence of the electron-donating or -withdrawing character of the substrates. These reagents led to 2-methyl-5-nitro-4-(tosylmethyl)-1,3-thiazole (**2b**) and 4-[(4-chlorophenylsulfonyl)methyl]-2-methyl-5-nitro-1,3-thiazole (**2c**), respectively (Table 1 entries 2 and 3). Similar good results were observed, which confirmed that the microwave-assisted method led to a more rapid and efficient synthesis of original sulfones.

**Table 1:** Classical heating method *versus* microwave-assisted synthesis of sulfones **2a** to **2c**.

Entry	Ar-	Product	Product number	Classical heating conditions ^a^		Microwave irradiation conditions ^b^	
				Time (h)	Yield (%)	Time (h)	Yield (%)
1	C_6_H_5_-	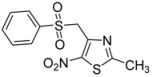	**2a**	24	84 [32]	0.5	96
2	*p*-CH_3_-C_6_H_4_-	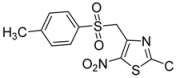	**2b**	24	57	0.5	86
3	*p*-Cl-C_6_H_4_-	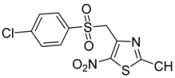	**2c**	24	69	0.5	76

^a^ This method was performed using 1 equivalent (equiv.) of 4-chloromethyl-2-methyl-5-nitro-1,3-thiazole (**1**) and 2 equiv. of sodium arylsulfinate derivative in anhydrous methanol (10 mL), under inert atmosphere (Ar) and 60 W lamp irradiation, at rt. ^b^ This method was performed using 1 equiv. of 4-chloromethyl-2-methyl-5-nitro-1,3-thiazole (**1**) and 2 equiv. of sodium arylsulfinate derivative in water (20 mL). An initial microwave irradiation of 200 W was used, the temperature being ramped up from r.t. to 100 °C and then held at 100 °C until the end of the reaction.

**Scheme 3:**
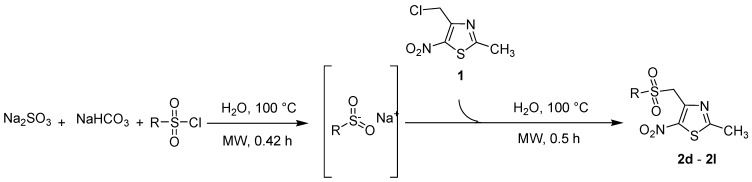
Preparation of sulfones **2d** to **2l**.

Next, to further diversify the chemical substituents on the sulfonyl group and to evaluate their influence on the antiproliferative activity of the corresponding products, these microwave-assisted operating conditions were used to synthesize new sulfonyl derivatives of **1** by reactions with various substituted sulfinate substrates. As such sulfinate salts are not commercially available, we performed the sodium-mediated reduction of sulfonyl chloride derivatives into the corresponding sulfinate anions, in aqueous conditions [46,47] and under microwave irradiation. Then, we investigated the above method using a mixture of sodium sulfite, sodium bicarbonate and sulfonyl chloride derivatives [48] and adapted it to the microwave methodology. Thus, the reduction of sulfonyl chloride derivatives was conducted with 3.4 equiv. of Na_2_SO_3_, 3.4 equiv. of NaHCO_3_, and 1 equiv. of the sulfonyl chloride derivative, in water at 100 °C, under microwave irradiation for 0.42 h. The compound **1** was directly added to the crude mixture, which was stirred for 0.5 h under the above conditions (MW 200 W, 100 °C) to give the corresponding sulfones **2d** to **2l** (Scheme 3) in moderate to good yields in a one-pot protocol (Table 2). 

**Table 2:** Microwave mediated preparation of several sulfones derivatives of 4-chloromethyl-2-methyl-5-nitro-1,3-thiazole (**1**).

R-	Product	Product number	Yield (%)
*p*-Br-C_6_H_4_-	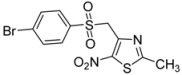	**2d**	68
*p*-F-C_6_H_4_-	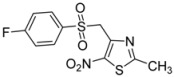	**2e**	82
*m*-F-C_6_H_4_-	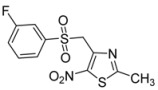	**2f**	65
*m*-CF_3_-C_6_H_4_-	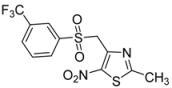	**2g**	71
*p*-CH_3_O-C_6_H_4_-	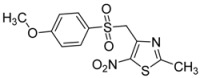	**2h**	60
*p*-C_2_H_5_-C_6_H_4_-	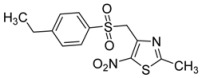	**2i**	31
CH_3_-	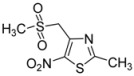	**2j**	52
2-bromothiophenyl-	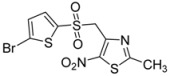	**2k**	58
2-naphthyl-	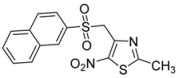	**2l**	90

All the reactions were performed using 2 equiv. of sulfonyl chloride, 3.4 equiv. of sodium sulfite, 3.4 equiv. of sodium carbonate in water (30 mL). An initial microwave irradiation of 200 W was used, the temperature being ramped up from r.t. to 100 °C, where it was held for 0.42 h. 1 equiv. of 4-chloromethyl-2-methyl-5-nitro-1,3-thiazole (**1**) was then added to the crude mixture, which was subsequently heated for 0.5 h*.*

**Scheme 4:**
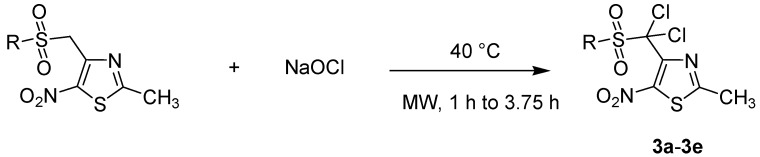
Preparation of dichlorinated sulfones **3a** to **3e**.

To assess the importance of the methyl group next to sulfonyl for the biological activity, we then evaluated a group of α-dichlorinated sulfonyl derivatives **3a** to **3e**. These compounds were prepared by reaction of sulfonyl derivatives with hypochlorite-based bleach (2.6% active chlorine) under lower microwave irradiation conditions (75 W, 40 °C) [49] (Scheme 4). Dichlorinated analogs were obtained in good yields (Table 3). 

**Table 3:** Microwave-mediated preparation of dichlorinated sulfone derivatives.

R-	Product	Product number	Yield (%)
C_6_H_5_-	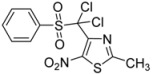	**3a**	81
*p*-CH_3_-C_6_H_4_-	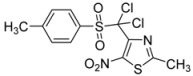	**3b**	61
-Cl-C_6_H_4_-	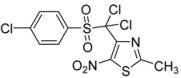	**3c**	68
*p*-Br-C_6_H_4_-	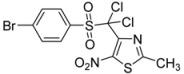	**3d**	79
*p*-F-C_6_H_4_-	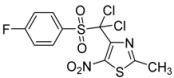	**3e**	88

All the reactions were performed using 1 equiv. of sulfonyl derivative (**2a** to **2f**) in 10 mL of sodium hypochlorite. A microwave irradiation of 75 W was used, the temperature being ramped up from r.t. to 40 °C, where the mixture was then held for 1 to 3.75 h.

**Figure 1:**
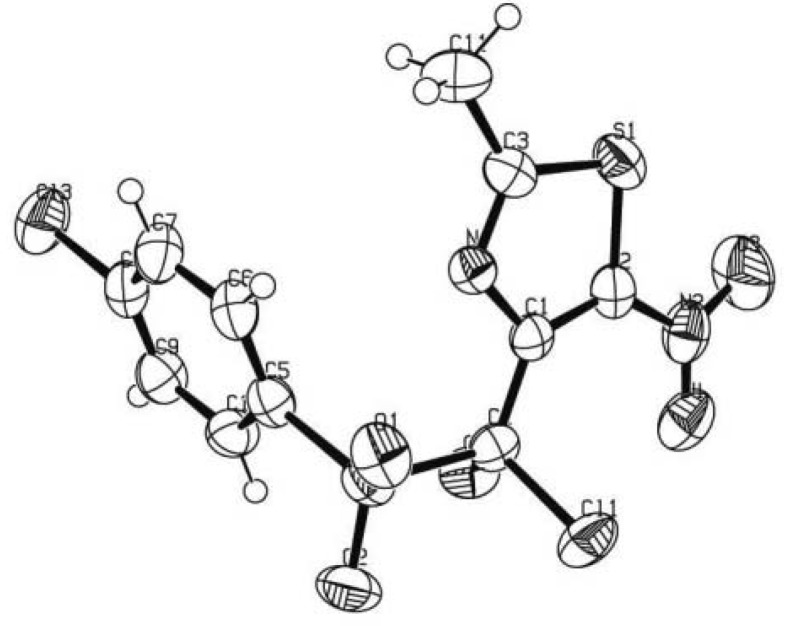
X-Ray structure of compound **3c**.

The structure of compound **3c** was unambiguously confirmed by X-ray structure analysis (Figure 1) (CCDC 908240). The other structures were assigned by analogy and spectral comparison.

### 2.2. *In Vitro* Biological Evaluation

The antiproliferative activity of the synthesized compounds was evaluated against two different cancer cell lines, CHO and HepG2, employing the MTT method [50] and doxorubicin as a reference drug. The cytotoxic activity *in vitro* was expressed as CC_50_ (µM), the concentration of compound that inhibits proliferation of cells by 50% as compared to untreated cells. The results of substance screening are summarized in Table 4.

All dihydrogenated sulfonyl derivatives displayed substantial antiproliferative activity towards HepG2 cells (7.7 µM ≤ HepG2 CC_50_ ≤ 25.6 µM) compared with doxorubicin used as reference drug (HepG2 CC_50_ = 0.2 µM), except compounds **2i** and **2j** for which no activity was observed on either of the cell lines. These data show that neither an elongation of the carbon chain at the *p*-position of the phenyl substituent nor an alkyl substituent on the sulfonyl group appears to favour the antiproliferative effects. 

**Table 4:** Antiproliferative activity of compounds **2a** to **3e**.

Product Number	Cancer cell toxicity ^a^ (µM)	
	CHO CC_50_	HepG2 CC_50_
**2a**	322.9 (± 4.66)	24.6 (± 0.78)
**2b**	237.3 (± 5.55)	7.7 (± 1.42)
**2c**	>62.5 ^c^	13.4 (± 1.47)
**2d**	>500 ^c^	11.7 (± 2.09)
**2e**	229.3 (± 4.02)	19.3 (± 1.21)
**2f**	321.1 (± 3.23)	23.6 (± 0.58)
**2g**	138.6 (± 2.64)	25.6 (± 2.13)
**2h**	136.8 (± 4.26)	20.6 (± 0.74)
**2i**	>500 ^c^	238.9 (± 2.27)
**2j**	>250 ^c^	>250 ^c^
**2k**	47.3 (± 2.28)	13.8 (± 1.07)
**2l**	106.2 (± 4.90)	8.5 (± 1.52)
**3a**	2.5 (± 0.23)	1.2 (± 0.09)
**3b**	1.2 (± 0.11)	1.0 (± 0.24)
**3c**	1.4 (± 0.06)	1.1 (± 0.17)
**3d**	1.3 (± 0.04)	1.2 (± 0.22)
**3e**	1.3 (± 0.04)	1.2 (± 0.34)
**Doxorubicin ^b^**	0.6	0.2

^a^ CC_50_ (µM) indicates the compound concentration that inhibits the proliferation of cells by 50% as compared to control untreated cells. The values are means ± SD of three independent experiments. ^b^ Doxorubicin was used as reference drug compound for cell toxicity. ^c^ No toxicity at the highest tested concentration.

Furthermore, this series was generally inactive on CHO cells, with CC_50_ values of between 47.3 and ≥500 µM, compared with doxorubicin (CHO CC_50_ = 0.6 µM). HepG2 is a commonly used human-derived hepatocarcinoma cell line expressing many of the hepatocyte-specific metabolic enzymes. The aim of this assay using HepG2 in addition to CHO cells was to evaluate the impact of metabolic activation of the tested compounds on cell viability [51]. Our results indicate that dihydrogenated sulfonyl derivatives, apart from **2i** and **2j**, need to be modified by a metabolic pathway to offer promising antiproliferative activity. Compounds **2b** and **2d** in particular displayed an antiproliferative effect 31 and 43 times higher respectively toward the HepG2 than toward the CHO cell line, which confirmed their high specificity for human liver tumor cells. 

Dichlorinated sulfones **3a** to **3e** were much more cytotoxic toward both the cell lines (1.0 µM ≤ CC_50_ ≤ 2.5 µM) than their dihydrogenated analogs, compared with doxorubicin used as reference of cellular toxicity. This result highlights the lack of cellular specificity of dichlorinated derivatives, confirming that the methyl group next to sulfonyl plays a key role in the antiproliferative activity of this series on human liver tumor cells.

## 3. Experimental

### 3.1. General

Melting points were determined on a Büchi B-540 and are uncorrected. Elemental analyses were carried out on an Interscience Flash EA 1112 series (Thermo Finnigan, San Jose, CA, USA) elemental analyzer at the Spectropole, Faculté des Sciences et Techniques de Saint-Jérome. Both ^1^H- and ^13^C-NMR spectra were determined on a Bruker Avance 200 spectrometer (operating at 200 MHz for ^1^H and 50 MHz for ^13^C). ^1^H and ^13^C-NMR shifts (δ) were reported in parts per million (ppm) with respect to CDCl_3_ 7.26 ppm for ^1^H and 77.0 ppm for ^13^C and DMSO-*d_6_* 2.50 for ^1^H and 39.7 ppm for ^13^C. Multiplicities were represented by s (singlet), d (doublet), t (triplet), q (quartet) and m (multiplet). Coupling constants (*J*) are in Hertz (Hz). The following adsorbent was used for column chromatography: silica gel 60 (Merck, Darmstadt, Germany, 230–400 mesh). Thin-layer chromatography was performed with Merck 60F-254 silica gel (0.25 mm layer thickness) in an appropriate solvent. All the reactions involving microwave instrumentation used the ETHOS Synth Lab station multimode reactor (Ethos Start, Milestone Inc., Rockford, IL, USA). The multimode microwave had a 25 twin magnetron (2 × 800 W, 2.45 GHz) with a maximum delivered power of 1,000 W in 10 W increments (pulsed irradiation). The multimode microwave featured a built-in magnetic stirrer (Teflon-coated stirring bar), direct temperature control of the reaction mixture with the aid of IR30 sensor on the reactor wall and software that enabled on line temperature control by regulation of microwave power output.

### 3.2. General Procedure for the Reaction of Compound ***1*** and Sodium Arylsulfinates to Synthesize Products ***2a*** to ***2c*** and Using Classical Heating Conditions

The corresponding sodium arylsulfinate (2 equiv.) was added to a solution of **1** (1 g, 5.2 mmol) in anhydrous methanol (10 mL). The reaction mixture was stirred at r.t., for 24 h, under an inert atmosphere (Ar) and 60 W lamp irradiation. After removal of the reaction mixture under reduced pressure, purification by chromatography on silica gel, elution with ethyl acetate and recrystallization from isopropanol (*i*-PrOH), the corresponding 4-arylsulfonylmethyl-2-methyl-5-nitro-1,3-thiazole was obtained.

### 3.3. General Procedure for the Reaction of Compound ***1*** and Sodium Arylsulfinates to Synthesize Products ***2a*** to ***2c*** and Using Microwave Irradiation

The corresponding sodium arylsulfinate (2 equiv.) was added to a solution of **1 **(1 g, 5.2 mmol) in water (20 mL). The reaction mixture was irradiated in a microwave oven (200 W, 100 °C, 0.5 h). A precipitate appeared and was filtered after cooling, washed with water (3 × 20 mL) and dried in a vacuum drying oven. Recrystallization from *i*-PrOH gave the corresponding sulfonyl derivative.

*2-Methyl-5-nitro-4-(tosylmethyl)-1,3-thiazole* (**2b**): Yellow solid; m.p. 179 °C (*i*-PrOH); ^1^H-NMR (CDCl_3_) δ: 2.43 (s, 3H, CH_3_), 2.70 (s, 3H, CH_3_), 5.02 (s, 2H, CH_2_), 7.31 (d, *J* = 7.9 Hz, 2H, 2 × CH), 7.68 (d, *J* = 7.9 Hz, 2H, 2 × CH); ^13^C-NMR (CDCl_3_) δ: 20.4 (CH_3_), 21.7 (CH_3_), 56.7 (CH_2_), 128.3 (2 × CH), 129.9 (2 × CH), 135.8 (C), 143.3 (C), 145.4 (C), 169.3 (C), C-NO_2_ not visible under these conditions; Anal. Calcd for C_12_H_12_N_2_O_4_S_2_: C, 46.14; H, 3.87; N, 8.97. Found: C, 46.41; H, 3.89; N, 9.07.

*4-[(4-Chlorophenylsulfonyl)methyl]-2-methyl-5-nitro-1,3-thiazole* (**2c**): Yellow solid; m.p. 180 °C (*i*-PrOH); ^1^H-NMR (CDCl_3_) δ: 2.70 (s, 3H, CH_3_), 5.04 (s, 2H, CH_2_), 7.51 (d, *J* = 8.8 Hz, 2H, 2 × CH), 7.76 (d, *J* = 8.8 Hz, 2H, 2 × CH); ^13^C-NMR (CDCl_3_) δ: 20.4 (CH_3_), 56.7 (CH_2_), 129.6 (2 × CH), 129.9 (2 × CH), 137.3 (C), 141.2 (C), 142.8 (C), 169.5 (C), C-NO_2_ not visible under these conditions; Anal. Calcd for C_11_H_9_ClN_2_O_4_S_2_: C, 39.70; H, 2.73; N, 8.42. Found: C, 39.95; H, 2.69; N, 8.55.

### 3.4. General Procedure for the Reaction of Compound ***1*** and Variously Substituted Sulfinate Salts to Synthesize Products ***2d*** to ***2l*** and Using Microwave Irradiation

Sodium sulfite (3.4 equiv.) and sodium bicarbonate (3.4 equiv.) were added to a solution of sulfonyl chloride (600 mg, 1 equiv.) in water (30 mL). The reaction mixture was irradiated in a microwave oven and reaction was carried out under irradiation at 100 °C at 200 W for 0.42 h. Then, compound **1** (300 mg, 1.56 mmol) was added *in situ*. The reaction mixture was irradiated for 0.5 h under the same conditions. After cooling down, the mixture was then extracted with chloroform (5 × 15 mL). The organic layers were dried over anhydrous sodium sulfate and removed under *vacuum*. Purification by column chromatography on silica gel, eluting with the appropriate solvent (**2d** and **2e**: CHCl_3_/EtOAc, 80/20; **2f**, **2g**, **2h**, **2l**: CHCl_3_/Et_2_O, 80/20; **2j**: EtOAc; **2k**: CHCl_3_/petroleum ether/EtOAc, 50/25/25) and recrystallization from *i*-PrOH gave the corresponding target product.

*4-[(4-Bromophenylsulfonyl)methyl]-2-methyl-5-nitro-1,3-thiazole* (**2d**): Yellow solid; m.p. 184 °C (*i*-PrOH); ^1^H-NMR (DMSO-*d_6_*) δ: 2.65 (s, 3H, CH_3_), 5.23 (s, 2H, CH_2_), 7.68 (d, *J* = 8.7 Hz, 2H, 2 × CH), 7.87 (d, *J* = 8.7 Hz, 2H, 2 × CH); ^13^C-NMR (DMSO-*d_6_*) δ: 20.1 (CH_3_), 56.2 (CH_2_), 128.8 (C), 130.3 (2 × CH), 132.6 (2 × CH), 138.0 (C), 143.3 (C), 170.4 (C), C-NO_2_ not visible under these conditions; Anal. Calcd for C_11_H_9_BrN_2_O_4_S_2_: C, 35.02; H, 2.40; N, 7.43. Found: C, 35.05; H, 2.34; N, 7.40.

*4-[(4-Fluorophenylsulfonyl)methyl]-2-methyl-5-nitro-1,3-thiazole* (**2e**): Yellow solid; m.p. 183 °C (*i*-PrOH); ^1^H-NMR (DMSO-*d_6_*) δ: 2.65 (s, 3H, CH_3_), 5.22 (s, 2H, CH_2_), 7.48 (m, 2H, 2 × CH), 7.83 (m, 2H, 2 × CH); ^13^C-NMR (DMSO-*d_6_*) δ: 20.1 (CH_3_), 56.3 (CH_2_), 116.8 (d, *J* = 23.5 Hz, 2 × CH), 131.6 (d, *J* = 7.0 Hz, 2 × CH), 135.1 (d, *J* = 4.7 Hz, C), 143.5 (C), 165.5 (d, *J* = 253.8 Hz, C-F), 170.4 (C), C-NO_2_ not visible under these conditions; Anal. Calcd for C_11_H_9_FN_2_O_4_S_2_: C, 41.77; H, 2.87; N, 8.86. Found: C, 41.75; H, 2.83; N, 8.85.

*4-[(3-Fluorophenylsulfonyl)methyl]-2-methyl-5-nitro-1,3-thiazole* (**2f**): Yellow solid; m.p. 154 °C (*i*-PrOH); ^1^H-NMR (DMSO-*d_6_*) δ: 2.64 (s, 3H, CH_3_), 5.27 (s, 2H, CH_2_), 7.57–7.69 (m, 4H, 4 × CH); ^13^C-NMR (DMSO-*d_6_*) δ: 20.1 (CH_3_), 56.0 (CH_2_), 115.3 (d, *J* = 24.3 Hz, CH), 121.7 (d, *J* = 20.3 Hz, CH), 124.6 (d, *J* = 3.4 Hz, CH), 132.0 (d, *J* = 8.0 Hz, CH), 140.8 (d, *J* = 7.0 Hz, C), 143.2 (C), 161.8 (d, *J* = 248.8 Hz, C-F), 170.4 (C), C-NO_2 _not visible under these conditions; Anal. Calcd for C_11_H_9_FN_2_O_4_S_2_: C, 41.77; H, 2.87; N, 8.86. Found: C, 41.36; H, 2.73; N, 8.67.

*2-Methyl-5-nitro-4-{[3-(trifluoromethyl)phenylsulfonyl]methyl}-1,3-thiazole* (**2g**): White solid; m.p. 121 °C (*i*-PrOH); ^1^H-NMR (DMSO-*d_6_*) δ: 2.61 (s, 3H, CH_3_), 5.33 (s, 2H, CH_2_), 7.86–8.32 (m, 4H, 4 × CH); ^13^C-NMR (DMSO-*d_6_*) δ: 20.0 (CH_3_), 56.0 (CH_2_), 123.4 (q, *J* = 273.0 Hz, CF_3_), 125.1 (q, *J* = 3.9 Hz, CH), 130.0 (q, *J* = 33.1 Hz, C-CF_3_), 131.2 (CH), 131.3 (q, *J* = 3.5 Hz, CH), 132.5 (CH), 139.9 (C), 143.2 (C), 146.6 (C), 170.5 (C); Anal. Calcd for C_12_H_9_F_3_N_2_O_4_S_2_: C, 39.34; H, 2.48; N, 7.65. Found: C, 39.40; H, 2.45; N, 7.54.

*4-[(4-Methoxyphenylsulfonyl)methyl]-2-methyl-5-nitro-1,3-thiazole* (**2h**): Brown solid; m.p. 154 °C (*i*-PrOH); ^1^H-NMR (DMSO-*d_6_*) δ: 2.66 (s, 3H, CH_3_), 3.85 (s, 3H, CH_3_), 5.12 (s, 2H, CH_2_), 7.12 (d, *J* = 7.2 Hz, 2H, 2 × CH), 7.64 (d, *J* = 7.2 Hz, 2H, 2 × CH); ^13^C-NMR (DMSO-*d_6_*) δ: 20.1 (CH_3_), 56.0 (CH_3_), 56.6 (CH_2_), 114.7 (2 × CH), 130.2 (C), 130.5 (2 × CH), 143.7 (C), 143.9 (C), 163.8 (C), 170.2 (C); *m/z* (EI): [M+H]^+^, found 329.0258. C_12_H_12_N_2_O_5_S_2_ requires 329.0260.

*4-[(4-Ethylphenylsulfonyl)methyl]-2-methyl-5-nitro-1,3-thiazole* (**2i**): White solid; m.p. 162 °C (*i*-PrOH); ^1^H-NMR (DMSO-*d_6_*) δ: 1.19 (t, *J* = 7.5 Hz, 3H, CH_3_), 2.65 (s, 3H, CH_3_), 2.70 (q, *J* = 7.5 Hz, 2H, CH_2_), 5.15 (s, 2H, CH_2_), 7.46 (d, *J* = 8.3 Hz, 2H, 2 × CH), 7.64 (d, *J* = 8.3 Hz, 2H, 2 × CH); ^13^C-NMR (DMSO-*d_6_*) δ: 15.3 (CH_3_), 20.1 (CH_3_), 28.3 (CH_2_), 56.4 (CH_2_), 128.3 (2 × CH), 128.9 (2 × CH), 136.1 (C), 143.7 (C), 151.2 (C), 170.2 (C), C-NO_2 _not visible under these conditions; *m/z* (EI): [M+H]^+^, found 327.0468. C_13_H_14_N_2_O_4_S_2_ requires 327.0468.

*2-Methyl-4-(methylsulfonylmethyl)-5-nitro-1,3-thiazole* (**2j**): Brown solid; m.p. 127 °C (*i*-PrOH); ^1^H-NMR (DMSO-*d_6_*) δ: 2.75 (s, 3H, CH_3_), 3.13 (s, 3H, CH_3_), 5.07 (s, 2H, CH_2_); ^13^C-NMR (DMSO-*d_6_*) δ: 20.2 (CH_3_), 41.7 (CH_3_), 54.6 (CH_2_), 144.4 (C), 170.7 (C), C-NO_2_ not visible under these conditions; *m/z* (EI): [M+Na]^+^, found 258.9815. C_6_H_8_N_2_O_4_S_2_ requires 258.9818.

*4-[(5-Bromothiophen-2-ylsulfonyl)methyl]-2-methyl-5-nitro-1,3-thiazole* (**2k**): Yellow solid; m.p. 167 °C (*i*-PrOH); ^1^H-NMR (DMSO-*d_6_*) δ: 2.68 (s, 3H, CH_3_), 5.31 (s, 2H, CH_2_), 7.45 (d, *J* = 3.9 Hz, 1H, CH), 7.56 (d, *J* = 3.9 Hz, 1H, CH); ^13^C-NMR (DMSO-*d_6_*) δ: 20.1 (CH_3_), 57.3 (CH_2_), 122.6 (C), 132.4 (CH), 136.5 (CH), 140.0 (C), 143.1 (C), 146.6 (C), 170.5 (C); Anal. Calcd for C_9_H_7_BrN_2_O_4_S_2_: C, 28.20; H, 1.84; N, 7.31. Found: C, 27.82; H, 1.76; N, 7.08.

*2-Methyl-4-[(naphtalen-2-ylsulfonyl)methyl]-5-nitro-1,3-thiazole* (**2l**): Yellow solid; m.p. 163 °C (*i*-PrOH); ^1^H-NMR (DMSO-*d_6_*) δ: 2.55 (s, 3H, CH_3_), 5.28 (s, 2H, CH_2_), 7.67–7.74 (m, 3H, 3 × CH), 8.09–8.17 (m, 2H, 2 × CH), 8.37 (d, *J* = 7.7 Hz, 1H, CH), 8.46 (d, *J* = 7.7 Hz, 1H, CH); ^13^C-NMR (DMSO-*d_6_*) δ: 20.0 (CH_3_), 56.5 (CH_2_), 123.4 (CH), 124.9 (CH), 127.2 (CH), 128.6 (CH), 128.8 (CH), 129.4 (CH), 131.1 (CH), 133.8 (CH), 136.1 (C), 143.3 (C), 146.5 (C), 170.2 (C), C-NO_2_ not visible under these conditions; Anal. Calcd for C_15_H_12_N_2_O_4_S_2_: C, 51.71; H, 3.47; N, 8.04. Found: C, 51.64; H, 3.48; N, 7.94.

### 3.5. General Procedure for the Dichlorination of Compounds ***2a*** to ***2e*** to Synthesize Products ***3a*** to ***3e*** Using Microwave Irradiation

The corresponding sulfone (1 equiv.) was added to a solution of hypochlorite-based bleach (2.6% active chlorine, 10 mL). The reaction mixture was irradiated in a microwave oven and reaction was carried out at 40 °C at 75 W from 1h to 3.75 h. After being cooled down, the mixture was then extracted with chloroform (3 × 20 mL). The organic layers were dried over anhydrous sodium sulfate and removed under *vacuum*. Purification by column chromatography eluting with CHCl_3_ and recrystallization from *i*-PrOH gave the corresponding required product.

*4-[Dichloro(phenylsulfonyl)methyl]-2-methyl-5-nitro-1,3-thiazole* (**3a**): Yellow solid; m.p. 169 °C (*i*-PrOH); ^1^H-NMR (CDCl_3_) δ: 2.76 (s, 3H, CH_3_), 7.57–7.80 (m, 3H, 3 × CH), 8.14–8.18 (m, 2H, 2 × CH); ^13^C-NMR (CDCl_3_) δ: 20.0 (CH_3_), 29.6 (C), 91.4 (C), 128.6 (2 × CH), 133.0 (2 × CH), 133.1 (C), 135.3 (CH), 143.2 (C), 165.5 (C); Anal. Calcd for C_11_H_8_Cl_2_N_2_O_4_S_2_: C, 35.98; H, 2.20; N, 7.63. Found: C, 36.04; H, 2.11; N, 7.39.

*4-[Dichloro(tosyl)methyl]-2-methyl-5-nitro-1,3-thiazole* (**3b**): Yellow solid; m.p. 165 °C (*i*-PrOH); ^1^H-NMR (CDCl_3_) δ: 2.49 (s, 3H, CH_3_), 2.75 (s, 3H, CH_3_), 7.39 (d, *J* = 8.2 Hz, 2H, 2 × CH), 8.03 (d, *J* = 8.2 Hz, 2H, 2 × CH); ^13^C-NMR (CDCl_3_) δ: 20.0 (CH_3_), 21.8 (CH_3_), 129.3 (2 × CH), 129.9 (C), 133.1 (2 × CH), 143.4 (C), 146.9 (C), 156.4 (C), 165.3 (C); C-NO_2_ not visible under these conditions; *m/z* (EI): [M+H]^+^, found 380.9532. C_12_H_10_Cl_2_N_2_O_4_S_2_ requires 380.9532.

*4-[Dichloro(4-chlorophenylsulfonyl)methyl]-2-methyl-5-nitro-1,3-thiazole* (**3c**): Yellow solid; m.p. 165 °C (*i*-PrOH); ^1^H-NMR (CDCl_3_) δ: 2.75 (s, 3H, CH_3_), 7.57 (d, *J* = 8.7 Hz, 2H, 2 × CH), 8.10 (d, *J* = 8.7 Hz, 2H, 2 × CH); ^13^C-NMR (CDCl_3_) δ: 20.0 (CH_3_), 91.4 (C), 129.0 (2 × CH), 131.7 (C), 134.4 (2 × CH), 142.6 (C), 143.3 (C), 165.7 (C); C-NO_2_ not visible under these conditions; Anal. Calcd for C_11_H_7_Cl_3_N_2_O_4_S_2_: C, 32.89; H, 1.76; N, 6.97. Found: C, 33.12; H, 1.70; N, 7.20.

C_11_H_7_N_2_O_4_S_2_, colorless prisms (0.25 × 0.15 × 0.1 mm^3^), MW = 401.66, orthorhombic, space group *P*21/c (T = 293 K), *a* = 15.6219 (1) Å, *b* = 9.6399 (3) Å, c = 20.5410 (5) Å, α = 90°, β = 90°, γ = 90°; *V* = 3093.34 (12) Å^3^, *Z* = 8, µ = 0.879 mm^−1^, *F*(000) = 1616, index ranges 0 ≤ *h* ≤ 22, 0 ≤ *k* ≤ 13, −29 ≤ *l* ≤ 0; θ range = 1.98–31.00°, 199 variables and 0 restraints, were defined for 4807 independent reflections with *I* ≥ 2σ(I) to *R1* = 0.0600, *wR2* = 0.1256, *GooF* = 1.052. CCDC 908240 contains the supplementary crystallographic data for this paper. These data can be obtained free of charge at www.ccdc.cam.ac.uk/data_request/cif of from the Cambridge Crystallographic Data Centre, 12, Union Road, Cambridge CB2 1EZ, UK; Fax: + 44 (1223) 336033; Email: deposit@ccdc.cam.ac.uk.

*4-[(4-Bromophenylsulfonyl)dichloromethyl]-2-methyl-5-nitro-1,3-thiazole* (**3d**): Yellow solid; m.p. 165 °C (*i*-PrOH); ^1^H-NMR (CDCl_3_) δ: 2.76 (s, 3H, CH_3_), 7.75 (d, *J* = 8.6 Hz, 2H, 2 × CH), 8.03 (d, *J* = 8.6 Hz, 2H, 2 × CH); ^13^C-NMR (CDCl_3_) δ: 20.1 (CH_3_), 91.3 (C), 131.3 (C), 132.0 (2 × CH), 132.2 (C), 134.4 (2 × CH), 143.3 (C), 165.7 (C); C-NO_2_ not visible under these conditions; Anal. Calcd for C_11_H_7_BrCl_2_N_2_O_4_S_2_: C, 29.61; H, 1.58; N, 6.28. Found: C, 29.27; H, 1.51; N, 5.97.

*4-[Dichloro(4-fluorophenylsulfonyl)methyl]-2-methyl-5-nitro-1,3-thiazole* (**3e**): Yellow solid; m.p. 145 °C (*i*-PrOH); ^1^H-NMR (CDCl_3_) δ: 2.76 (s, 3H, CH_3_), 7.26–7.32 (m, 2H, 2 × CH), 8.17–8.23 (m, 2H, 2 × CH); ^13^C-NMR (CDCl_3_) δ: 20.1 (CH_3_), 91.4 (C), 116.1 (d, *J* = 23.4 Hz, 2 × CH), 129.0 (d, *J* = 3.2 Hz, C-F), 136.1 (d, *J* = 10.3 Hz, 2 × CH), 143.3 (C), 164.5 (C), 165.6 (C), 169.6 (C); *m/z* (EI): [M+H]^+^, found 384.9280. C_11_H_7_FCl_2_N_2_O_4_S_2_ requires 384.9281.

### 3.6. *In Vitro* Biological Evaluation

*In Vitro* Cytotoxicity Evaluation on CHO and HepG2 Cell Lines

CHO and HepG2 cell lines were maintained at 37 °C, 6% CO_2_, 14% O_2_, 80% N_2_, with 90% humidity in RPMI supplemented with 10% fœtal bovine serum, 1% L-glutamine (200 mM) and penicillin (100 U/mL) / streptomycin (100 µg/mL) (complete RPMI medium). 

*In vitro* cytotoxicity evaluation on CHO and HepG2 cell lines was performed according to the method described by Mosmann [50] with slight modifications. Briefly, 5 × 10^3^ cells in 100 µL of culture medium (RPMI + 10% CO_2_) were inoculated into each well of 96-well plates and incubated at 37 °C in a humidified 6% CO_2_, 14% O_2_, 80% N_2_ atmosphere. After 24 h incubation, 100 µL of medium with various product concentrations was added and the plates were incubated from 24 h (CHO) to 72 h (HepG2). Duplicate assays were performed for each sample. At the end of the treatment and incubation, the medium was aspirated from the wells and 10 µL yellow MTT (3-(4,5-dimethyl-2-thiazolyl)-2,5-diphenyl-2*H*-tetrazolium bromide) solution (5 mg MTT/mL in PBS) was added to each well with 100 µL of medium without fœtal bovine serum. Cells were incubated for 2 h at 37 °C to allow MTT oxidation by mitochondrial dehydrogenase in the viable cells. After 2 h, the MTT solution was aspirated and DMSO (100 µL) was added to each well to dissolve the resulting blue formazan crystals. Plates were then shaken vigorously (300 rpm) for a few minutes. The absorbance was measured at 570 nm with 630 nm as reference wavelength, using a microplate spectrophotometer. DMSO was used as blank and doxorubicin as positive control. 

Cell viability was calculated as percentage of control (cells incubated without compound). The 50% cytotoxic concentrations (CHO CC_50 _and HepG2 CC_50_) were determined by non-linear regression analysis processed on dose-response curves, using the Table Curve software 2D v.5.0. CC_50_ values represent the mean value calculated from three independent experiments.

## 4. Conclusions

We have developed an efficient, rapid and eco-friendly microwave-based method for synthesizing 4-alkyl- and 4-arylsulfonylmethyl-2-methyl-5-nitro-1,3-thiazoles by the reaction of 4-chloromethyl-2-methyl-5-nitro-1,3-thiazole (**1**) with various substituted sodium sulfinates, either commercially available or obtained from sulfonyl chlorides previously reduced by a sodium-mediated reaction in aqueous medium. 

Biological evaluation of these synthesized compounds revealed the promising antiproliferative activity toward HepG2 cell line of most of the dihydrogenated sulfonyl derivatives after metabolic activation. Their dichlorinated analogs were synthesized using hypochlorite-based bleach (2.6% active chlorine) under microwave irradiation. Biological results showed that these compounds were much more cytotoxic toward both cell lines, showing their lack of cellular specificity and confirming that the methyl group next to sulfonyle played a key role in the antiproliferative activity of this series on human liver tumor cells. 

These promising results suggest that further research should be done on 4-arylsulfonylmethyl-2-methyl-5-nitro-1,3-thiazoles as potential anticancer agents in the treatment of liver cancer.
